# Therapies for Meibomian Gland Dysfunction: A Systematic Review and Meta-Analysis of Randomized Controlled Trials

**DOI:** 10.2174/1874364101711010346

**Published:** 2017-11-22

**Authors:** Kannan Sridharan, Gowri Sivaramakrishnan

**Affiliations:** 1 Department of Pharmacology and Therapeutics, College of Medicine and Medical Sciences, Arabian Gulf University, Manama, Bahrain.; 2 Department of Oral Health, Fiji National University, Suva, Fiji

**Keywords:** Antimicrobials, Heat therapy, MGD, Omega-3-Fatty Acids, Chronic, RCT

## Abstract

**Introduction:**

Meibomian Gland Dysfunction (MGD) is a common, often overlooked, chronic condition affecting eyes for which various therapies are being evaluated. Considering the absence of a systematic review and meta-analysis, the present review was carried out.

**Methods:**

An appropriate search strategy eligibility criteria were framed and electronic databases were scrutinized for appropriate literature. Randomized Controlled Trials (RCTs) enrolling patients diagnosed with MGD were included. Outcome measures were Tear Break Up Time (TBUT), Schirmer’s test, Meibomian Gland (MG) secretion score, MG plugging score, OSDI and SPEED. Cochrane’s tool was used to assess the risk of bias and Forest plot were generated either with fixed or random effects model, with Standardized Mean Difference (SMD).

**Results:**

TBUTs, Schirmer’s test and OSDI scores for systemic antimicrobials with placebo were 1.58 [1.33, 1.83], 2.93 [0.78, 5.09] and -3.58 [-4.28, -2.89] respectively. No quantitative synthesis was attempted for either mebiomian plugging or meibomian secretion scores and no significant changes were observed with any other outcome parameter.

**Conclusion:**

Only the systemic antimicrobials were found to improve the clinical features of meibomian gland dysfunction. Varying effects of different therapeutic agents (heat therapies, omega-3-fatty acids and castor oil) were identified for MGD but the risk of bias pertaining to randomization and allocation concealment was found to be associated with most of the current RCTs. More high quality evidence is required to confirm the findings of the present review.

## 1 INTRODUCTION

Meibomian Gland Dysfunction (MGD), a term coined by Korb and Henriques in 1980, is a chronic condition affecting the meibomian gland resulting in decreased secretion or poor quality of meibum [1]. MGD is widely prevalent worldwide ranging between 3.5 and 70% [2-6]. MGD can be asymptomatic (identified through meibography) or can present as thickened and telangiectatic lid margin often associated with either madarosis or trichiasis [7]. The traditional treatments of MGD comprise warm compression and maintaining appropriate hygiene of the eyelids by removing the obstructed meibum. In addition, antimicrobial and anti-inflammatory agents are also prescribed [8]. Recently, intraductal meibomian gland probing, novel heat therapies, N-acetyl-cysteine, omega-3 essential fatty acids and cyclosporine A have been debated to have clinical utility in MGD [9]. Report by the clinical trials subcommittee of international workshop on meibomian gland dysfunction in 2009 concluded that many of the methodological features of the published trials in patients with MGD were not satisfactory [10]. Considering the lacunae of a systematic review or meta-analysis, the present review was carried out to assess the efficacy of various therapeutic options available for patients with MGD that have been evaluated through randomized clinical trials.

## 2 METHODS

### 2.1 Information Sources and Search Strategy

The protocol for this review was registered with International prospective register of systematic reviews (PROSPERO) with the registration number CRD42016035704. The review protocol can be accessed at http://www.crd.york.ac.uk/PROSPERO/display_record.asp?ID=CRD42016035704. A thorough literature search was conducted and was completed on 15 January 2017. The primary data base used was Medline (*via* PubMed), Cochrane central register of clinical trials (CENTRAL) and Database of Abstracts of Reviews of Effects (DARE). The key words used were meibomian gland disease [tiab], tarsal gland disease [tiab], posterior blepharitis [tiab] and meibomian gland dysfunction [tiab]. This search was further supplemented by hand searching of relevant references from review articles and other eligible studies. No limits were applied to the year of study but only studies published in English language were included in the present review.

### 2.2 Eligibility Criteria

Only those studies with randomized controlled design with the following requirements were included in the present study. Studies that enrolled participants diagnosed with MGD clinically, with or without meibography, wherein the study participants have been randomized to receive at least one of the two interventions including placebo were considered. Studies enrolling patients with contact lenses or had undergone any ocular surgery in the past were excluded from the review. The outcome measures were Tear Break-Up Time (TBUT), Schirmer’s test, meibomian gland secretion score, meibomian gland plugging score, Standard Patient Evaluation of Eye Dryness (SPEED) and Ocular Surface Disease Index (OSDI) scores.

### 2.3 Study Procedure

Both the authors independently screened the data bases and independently reviewed the identified abstracts for suitability. Full-text articles were obtained following abstract screening for those found to be eligible to be included in the review. A pre-tested data extraction form was created and both the authors independently extracted the following data from each eligible study: trial site, year, trial methods, participants, interventions, and outcomes. Disagreement between the authors was resolved through discussion. The extracted data were analysed using non-Cochrane mode in RevMan 5.3 software. The methodological quality of eligible trials was independently assessed by both the authors using the Cochrane collaboration’s tool for assessing the Risk of Bias. We followed the guidance to assess whether trials took adequate steps to reduce the risk of bias across six domains: sequence generation, allocation concealment, blinding (of participants, personnel, and outcome assessors), incomplete outcome data, selective outcome reporting, and other sources of bias. The judgment was categorized into low, high or unclear risk of bias using Risk Of Bias (ROB) tool [11]. Mean Difference (MD) was used as the pooled estimate for the continuous outcome measures and 95% confidence interval (95% CI) was used to represent the deviation from the point estimate. The heterogeneity between the studies was assessed using Forest plot visually, I^2^ statistics wherein more than 30% was considered to have moderate to severe heterogeneity and Chi-square test with a statistical P-value of less than 0.10 to indicate statistical significance [11]. When substantial heterogeneity was observed, the clinical or methodological differences between the studies which can be accounted for the heterogeneity were ruled out before applying the principles of meta-analysis. Random-effects model was used in cases of moderate to severe heterogeneity except in case of effect estimates of the individual studies lying in different directions. Considering the presence of only few eligible trials for each of the assessed outcome measures, publication bias could not be assessed. The present meta-analysis was conducted and presented in accordance with Preferred Reporting Items for Systematic Reviews and Meta-Analyses (PRISMA) guidelines [12]. The grading of quality of included studies was carried out as per Cochrane’s Grading of Recommendations Assessment, Development and Evaluation tool (GRADE) [13].

## 3 RESULTS

### 3.1 Search Strategy

The above mentioned search strategy led a hit of 394 articles. Fig. (**1**) illustrates the study flow chart as per PRISMA guidelines. A total of 22 studies [14-35] were identified eligible for the present review. Of these, two [14, 15] presented data in the format not subjected to quantitative synthesis and so were not included for the meta-analysis. Supplementary Table **1** describes the key study details of the 22 eligible studies and Fig. (**2**) depicts the risk of bias of the included studies in the meta-analysis as per Cochrane risk of bias tool. A wide variety of interventions were compared for the management of MGD such as anakinra, topical cyclosporine, different heat devices, systemic antimicrobials (minocycline, azithromycin, doxycycline), N-acetyl cysteine, betamethasone-sulfacetamide, loteprednol, omega-3-fatty acids, castor oil and tobramycin-dexamethasone.

### 3.2 Pooled Results

#### 3.2.1 TBUT

**Fig. (5):**
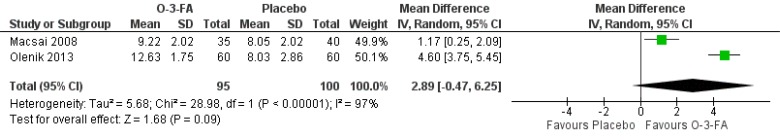
Forest plot of TBUT of studies comparing omega-3-fatty acids (O-3-FA) with placebo.

A total of four studies compared TBUT (s) between different heat therapies in 450 participants. Pooling of the effect estimates from individual studies could not be performed because of the moderate to severe heterogeneity observed between the studies and different directions of the individual effect estimates. Fig. (**3**) depicts the pooled analysis of TBUT of two systemic antimicrobials (one each with minocycline and doxycycline) with placebo and was favoring the antimicrobials with a MD of 1.58 [1.33, 1.83]. Two studies compared the efficacy of N-acetylcysteine, one each against placebo and betamethasone-sulfacetamide on TBUT. MD of both the individual studies and pooled analysis were not statistically significant as depicted in Fig. (**4**). Only one each compared the effect of topical corticosteroids, topical cyclosporine, castor oil, topical anakinra and two systemic antimicrobials (oral azithromycin and doxycycline) and therefore, pooling of the results was not possible. Two studies compared the effect of omega-3-fatty acids with placebo on TBUT with the pooled MD of 2.89 [-0.47, 6.25] (Fig. **5**).

#### 3.2.2 Schirmer’s Test

Two studies compared the effect of different heat therapies with placebo on Schirmer’s test and the pooled estimate was not statistically significant with MD of -2.46 [-5.93, 1.01]. Similarly, no significant difference was obtained in the pooled analysis of study results evaluating N-acetylcysteine with MD of -0.78 [-3.67, 2.11]. Fig. (**6**) depicts the Forest plot of pooled analysis of studies comparing the different systemic antimicrobials with placebo on Schirmer’s test and was found to favor antimicrobials with a pooled MD of 2.93 [0.78, 5.09]. Systemic antimicrobials have been found to have large therapeutic effect in comparison to placebo in improving the outcomes of Schirmer’s test.

#### 3.2.3 Meibomian Gland Secretion Score

One each compared one form of heat therapy, systemic antimicrobials, topical corticosteroids, omega-3-fatty acids with placebo on Meibomian gland secretion score and so meta-analysis was not attempted for this outcome measure.

#### 3.2.4 Meibomian Gland Plugging Score

One each compared the effect of topical cyclosporine, systemic antimicrobials and castor oil on meibomian gland plugging scores in comparison with placebo and meta-analysis was not attempted.

#### 3.2.5 OSDI and SPEED Scores

Two studies comparing the heat therapy with placebo evaluated the effect on OSDI scores with MD of -4.54 [-18.41, 9.34] and was not statistically significant. On the contrary, two other studies compared the effect of systemic antimicrobials with placebo on OSDI scores and the pooled estimate was found to be statistically significant -3.58 [-4.28, -2.89]. Systemic antimicrobials also have large therapeutic effect in comparison to placebo in improving the patient reported symptoms scores.

## DISCUSSION AND CONCLUSION

The present study is as an attempt to systematically synthesize the available high quality evidence for the management of MGD. We included randomized controlled trials that assessed the effect of various heat therapies, topical applications of corticosteroids, cyclosporine, anakinra, castor oil, topical and systemic antimicrobial agents, N-acetyl cysteine and omega-3-fatty acids. We found a significant improvement in TBUT, Schirmer’s test and patient satisfactory scores with systemic antimicrobials. Pooled analysis cannot be attempted for outcome measures such as meibomian gland secretion score and meibomian gland plugging score.

MGD is a chronic condition that is often overlooked and is characterized by inflammation, swelling and abnormal secretion, leading to dry eyes. Various treatment options have been evaluated for appropriate management of MGD. Depending on the severity of the disease condition, MGD has been classified into four grades [36]. Conservative measures such as lid warming either through digital massage or through heat appliances are advised during the initial phase followed by several medications in case of non-responders or severe stages of the disease. Although profound evidence exists on various therapeutic options for managing MGD, they are not completely dependable. No uniform way of defining the study population, non-standard diagnostic technique and inadequate quality and consistency of the studies have been some of the major drawbacks identified by the clinical trials subcommittee of “The international workshop on meibomian gland dysfunction” regarding treatment strategies for MGD [37]. Similarly in the present review we identified that critical items related to randomization such as method of randomization and allocation concealment were poorly expressed in the studies. Further not much uniformity has been noted in terms of analysis and expression of the change in outcome measures creating disparity between studies. Hence, there is a clear cut need for improving various aspects of the studies evaluating therapeutic options for MGD. Although a similar traditional review [38] exists in the past, the present one is a network meta-analysis summing up the current evidence on the available treatment strategies.

The study is limited by the fact that we did not assess the publications from EMBASE, proportion of patients attaining complete cure and the exploratory nature of pooled results due to small number of studies in each outcome in the present review. Also, we found only few numbers of eligible studies to be included for almost all the outcome measures. To conclude heat therapies, only systemic antimicrobials were found to have some usefulness in the management of MGD. Varying effects of different therapeutic agents (heat therapies, omega-3-fatty acids and castor oil) were identified for MGD but the risk of bias pertaining to randomization and allocation concealment was found to be associated with most of the current RCTs. However, more high quality studies are needed to confirm the findings of individual treatment options available for managing MGD.

## Figures and Tables

**Fig. (1):**
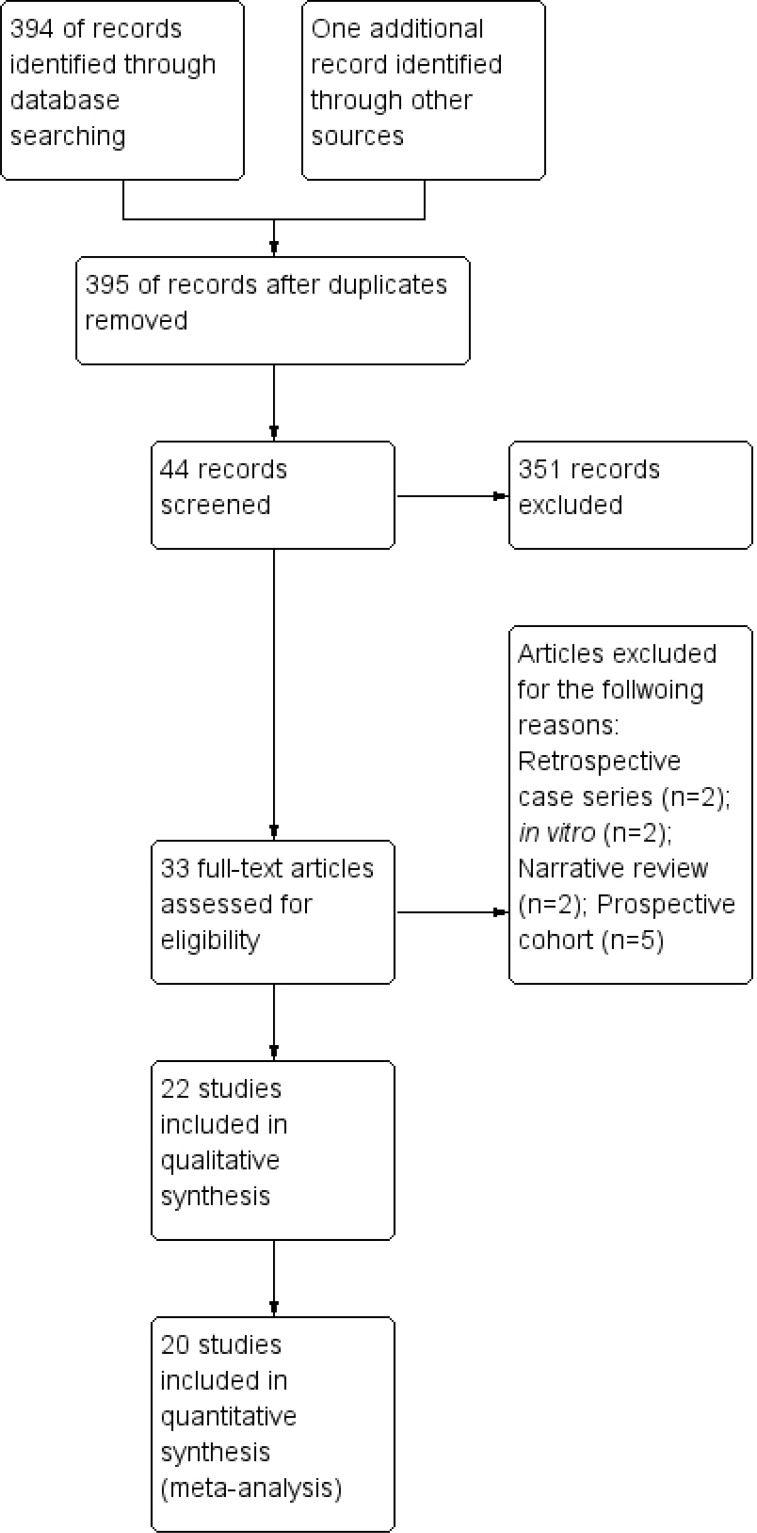
Study flow chart as per PRISMA template.

**Fig. (2):**
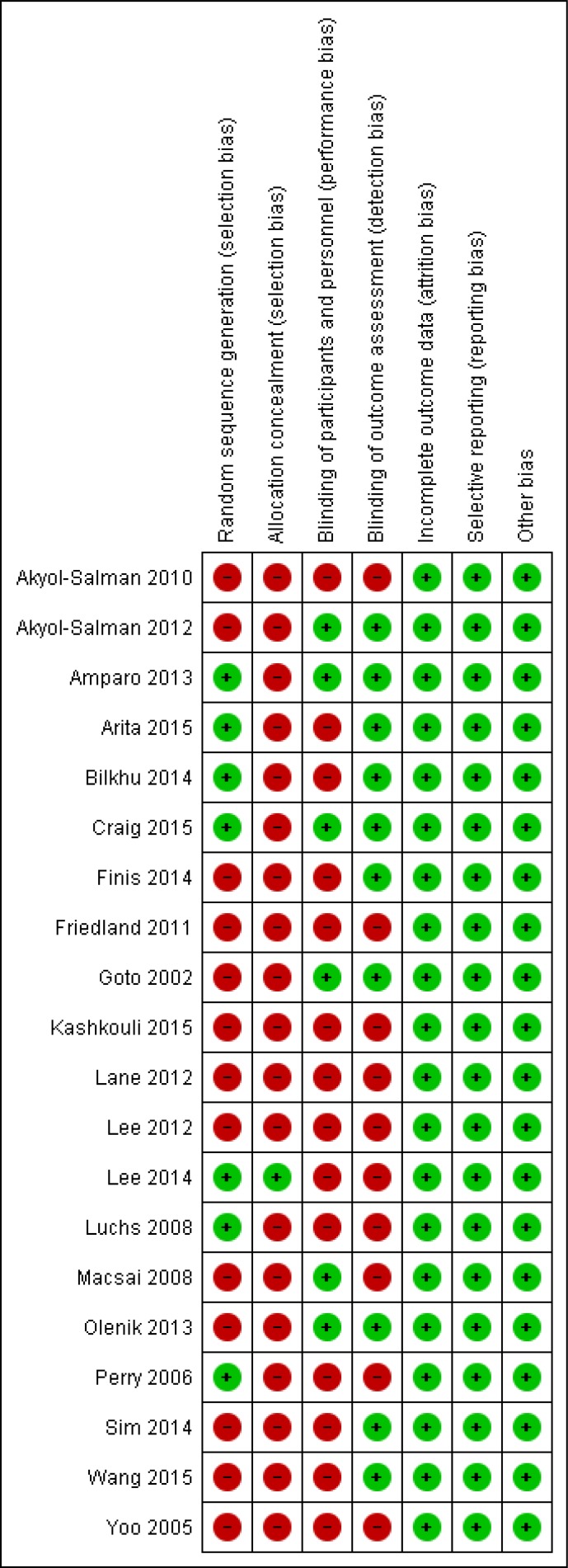
Risk of bias graph of the included studies in meta-analysis.

**Fig. (3):**
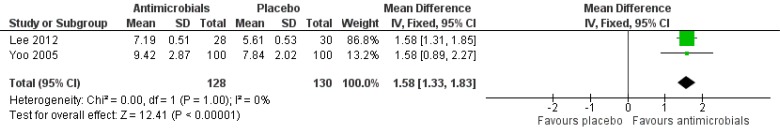
Forest plot of TBUT of systemic antimicrobials with placebo.

**Fig. (4):**
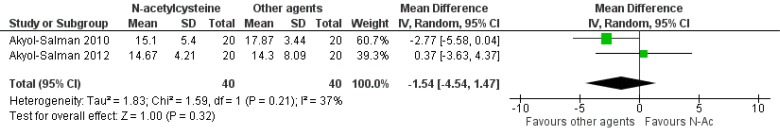
Forest plot of TBUT of N-acetylcysteine with other agents.

**Fig. (6):**
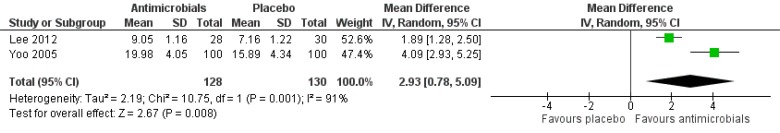
Forest plot of studies comparing systemic antimicrobials with placebo on Schirmer’s test.
