# Supplementary material for: Therapies for Meibomian Gland Dysfunction: A Systematic Review and Meta-Analysis of Randomized Controlled Trials
Source: Open Ophthalmol J. 2017 Nov 22;11:346–54. doi: 10.2174/1874364101711010346 (PMC5730954; doi:10.2174/1874364101711010346)
Supplement: Supplementary file 1 [file TOOPHTJ-11-346_SD1.pdf]

Send Orders for Reprints to reprints@benthamscience.ae

The Open Ophthalmology Journal, 2017, *11*, i-iv

BENTHAM OPEN

CrossMark

The Open Ophthalmology Journal

Supplementary Material

Content list available at: www.benthamopen.com/TOOPHTJ/

DOI: 10.2174/1874364101711010346

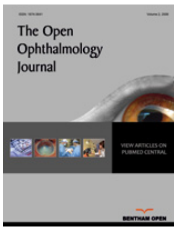

Therapies for Meibomian Gland Dysfunction: A Systematic Review and Meta-Analysis of Randomized Controlled Trials

Kannan Sridharan[1,*] and Gowri Sivaramakrishnan[2]

[1]*Department of Health Sciences, Fiji National University, Suva, Fiji*
[2]*Department of Oral Health, Fiji National University, Suva, Fiji*

Supplementary Table 1. Summary of the key details of the included studies in the meta-analysis.

**Study id; year; country**	**Population**	**Intervention**	**Comparator**	**Outcome**
Prabhasawat *et al* [14]; 2012; Thailand	Patients with MG obstruction, abnormal secretion, and/or lid margin inflammation, TBUT ≤ 8 seconds in each eye with at least one of the symptoms of tear film instability, irritation, photophobia, and tearing were included; Those with severe ocular surface abnormalities, systemic or ocular disorders such as ocular surgery, glaucoma or contact lens wearer, ophthalmic infections or immunosuppressed were excluded	0.05% cyclosporine A eye drops twice daily for 3 months	0.5% carboxy methyl cellulose eye drops twice daily for 3 months	OSDI, lid margin inflammation, meibomian gland expression, conjuctival injection, corneal and interpalpebral dye staining, noninvasive TBUT and Schirmer's test
Rubin *et al* [15]; 2006; USA	Diagnosed as MGD with posterior lid erythema and MG telangiectasia; Patients with punctal occlusion and those who have received oral doxycycline or corticosteroids, systemic diseases, pregnant or lactating women were excluded from the study	Cyclosporine ophthalmic emulsion 1 drop every 12 hours for 12 weeks	Tobramycin 0.3%/ 0.1% Dexamethasone 0.1% one drop 12 hours for 12 weeks	Slit-lamp examination, intra-ocular pressure measurement, TBUT, Schirmer's test, MG secretion score, tear lysozyme levels and eyelid health parameters
Amparo *et al* [16]; 2013; USA	Patients aged ≥ 18 years with symptoms of dry eye disease with positive corneal or conjuctival epithelial staining in either eye on the modified Oxford grading scale and diagnosis of MGD, normal eyelid position and closure, and negative urine pregnancy test; Those with eyelid surgery, treatment with anakinra, ocular surgery, active ocular infection or allergies, use of isotretinoin and corneal epithelial defect > 1 mm^2 were excluded.	Anakinra 2.5% one drop thrice daily for 12 weeks	1% carboxy methyl cellulose eye drops thrice daily for 12 weeks	OSDI, TBUT, Schirmer's test, corneal fluorescence staining, meibomian secretion quality and adverse effects
Perry *et al* [17]; 2006; USA	Patients ≥ 18 years with slit-lamp diagnosis of MGD and OSDI ≥ 12; Individuals with contact lens, active ocular diseases or ocular surgery in the past 3 months, ocular allergies, autoimmune disease, pregnant women, lactating mothers, isotretinoin use in the past 6 months or hypersensitive to cyclosporine A were excluded	Topical cyclosporine one drop in each eye twice a day for 3 months	Topical placebo one drop in each eye twice a day for 3 months	TBUT, Schirmer's test, total ocular symptoms score, meibomian gland inclusions and Lissamine Green staining score
Wang *et al* [18]; 2015; New Zealand	Patients aged 18 years and above with mild to moderate symptoms of dry eye without the use of contact lens in the past 48 hours, without any systemic medications and systemic diseases affecting eye were included in the study	Eye mask for 10 minutes	Microwave-heated flaxseed eye bag for 10 minutes	TBUT, Tear meniscus height, tear film lipid layer grade, treatment preference and outer and inner eyelid temperatures

Suppl. table 1 contd.....

Study id; year; country	Population	Intervention	Comparator	Outcome
Lee *et al* [19]; 2012; Korea	Patients with stage 3 or 4 MGD were included and those with history of intra-ocular surgery/ocular infection/autoimmune disease or topical or oral antibiotics, cyclosporine A, corticosteroids or non-steroidal anti-inflammatory drugs, current punctual occlusion, pregnancy, lactating women and children were excluded	50 mg minocycline orally twice a day with 0.1% sodium hyaluronate eye drops 4 times a day for 2 months	0.1% sodium hyaluronate eye drops 4 times a day for 2 months	TBUT, Schirmer's test, MG secretion score, OSDI score, tear cytokine levels and biomicroscopic examination of lid margins
Arita *et al* [20]; 2015; Japan	Patients diagnosed as obstructive MGD with ocular symptoms of at least one lid margin abnormality; Those with ocular allergies, contact lens, eye surgery or systemic diseases were excluded	Two non-wet and three wet interventions were tested in this study of which only the non-wet intervention (Azuki no Chikara) is considered for evaluation in this review. The non-wet intervention was a warming eye pillow containing red beans that were warmed in a microwave oven	No intervention	TBUT, Schirmer's test, visual analog scale for ocular discomfort, eyelid skin, central corneal and tarsal conjuctival temperatures, meibomian gland area, meibum grade, superficial punctate keratopathy score
Finis *et al* [21]; 2014; Germany	Patients aged 18 years and above with SPEED score ≥ 8, lipid layer thickness ≤ 61 and expressible MG secretion score of ≤ 4 were included; Those with tetracyclines, antihistamines, isotretinoin within the past 3 months or topical cyclosporine A in the past one month, ocular surgery or trauma within the past 3 months, eyelid abnormalities and systemic diseases resulting in dry eye were excluded	A 12 minute Lipiflow© single time treatment	Lid warming and massage twice daily for 3 months	Schirmer's test, SPEED and OSDI scores, tear film osmolarity, tear meniscus height, ocular surface staining, lid margin parallel conjuctival folds, TBUT, lipid layer thickness, and number of expressible meibomian glands
Bilkhu *et al* [22]; 2014; UK	Patients aged 18 years and above with OSDI ≥ 12, presence of MG orifice plugging, TBUT < 10s, Schirmer's test > 5.5 mm after 5 minutes	Heated eye bag (40 seconds in microwave on full power) MGDRx© 5 minutes twice a day for 2 weeks	Non-heated eye bag 5 minutes twice a day for 2 weeks	MG secretion score, OSDI score, visual acuity, corneal topography, conjuctival hyperemia, meibomian gland quality, meibomian gland expressibility, drop-out, meibomian gland osmolarity, tear film stability, meniscus height and ocular surface staining
Luchs *et al* [23]; 2008; USA	Patients with posterior blepharitis with moderate degree of either redness or swelling were included; Those with lid structural abnormalities, inflammation or active structural change in the iris/anterior chamber, ocular fungal or viral infections, intra-ocular surgery, herpes keratitis or glaucoma were excluded	Azithromycin eye drops one drop twice a day for 2 days and once a day for 12 days with warm compress for 5-10 min twice a day for 2 weeks	Warm compress 5-10 min twice a day for 2 weeks	Lid debris, lid redness, lid swelling, MG plugging, quality of MG secretions, overall ocular symptoms relief score
Lane *et al* [24]; 2012; USA	Patients aged 18 years and above with dry eye symptoms, SPEED score of at least 6 and evidence of MG obstruction were included; Those with evidence of ocular surgery or trauma within the past 3 months, ocular surface abnormality compromising the corneal integrity in either eye, eyelid abnormalities or systemic disease were excluded	Lipiflow© 12 min single time office treatment	iHeat© warm compresses 5 minutes for at least 10 days	TBUT, MG secretion score, number of glands secreting any liquid and number of glands secreting clear liquid, SPEED and OSDI scores, device related adverse events, discomfort/pain score, best spectacle corrected visual acuity and, corneal and conjuctival stainings

Suppl. table 1 contd.....

Study id; year; country	Population	Intervention	Comparator	Outcome
Friedland *et al* [25]; 2011; USA	Patients aged 18 years and above with self-reported dry eye symptoms, need for regular use of artificial tears, lubricants, or rewetting drops in both eyes, evidence of meibomian gland obstruction in the central five glands, and a minimum SPEED score of 6 for each eye; Those with recent ocular trauma or surgery or herpetic keratitis or blepharitis or meibomitis, Steven-Johnson syndrome, Riley Day syndrome, sarcoidosis, leukemia, pregnant women or lactating women were excluded	Automated device containing a lid warmer for 12 min single time with warm compression through hand held instrument for one eye in each of the study participants	Automated device containing a lid warmer for 12 min single time	TBUT, corneal staining, meibomian gland secretion score, ocular health examination, patients discomfort or pain evaluation, time to complete treatment, and SPEED and OSDI scores
Craig *et al* [26]; 2015; New Zealand	Patients with mild to moderate signs of MGD were included and those with clinical skin treatments in the past 2 months or implants beneath the treatment area and those wearing contact lenses were excluded	Intense pulsed therapy at 1, 15 and 45 days. Four pulses at an intensity ranging between 9 and 13 J/cm^2 were applied	Placebo at 1, 15 and 45 days	Best spectacle corrected visual acuity, Lipid layer grade, TBUT, tear evaporation rate, tear meniscus height, visual analog scale for bulbar conjuctival injection, SPEED and OSDI score
Akyol-Salman *et al* [27]; 2012; Turkey	Patients with diagnosis of MGD without any systemic abnormalities such as hypertension, ocular surgery or allergic reaction and not having used any therapies for MGD	5% N-acetylcysteine 4 times a day for 1 month	0.1% betamethasone/10% sulfacetamide 4 times a day for 1 month	Slit-lamp examination, intraocular pressure measurement, TBUT and Schirmer's test
Akyol-Salman *et al* [28]; 2010; Turkey	Patients with slit-lamp diagnosis of MGD as evidenced by thickening or irregularity of the eye lid margins; erythema of posterior lid margin; telangiectasis were included. Those with systemic abnormalities, previous ocular surgery, intraocular pathology or history of allergy were excluded	5% topical N-acetyl cysteine 4 times a day for 1 month	Preservative free artificial tears 4 times a day for 1 month	Slit-lamp examination, intraocular pressure measurement, TBUT, Schirmer's test, ocular symptoms
Lee *et al* [29]; 2014; South Korea	Patients with moderate to severe MGD were included; those with ocular surgery, glaucoma, ocular hypertension, ocular infection/allergy, autoimmune diseases, contact lens wearers, pregnant, lactating women or those with current punctual occlusion were excluded	Topical loteprednol with warm compresses 4 times a day for 2 months	Warm compress 2 times a day for 2 months	TBUT, MG secretion score, OSDI score, tear cytokine levels
Olenik *et al* [30]; 2013; Spain	Patients between 18 and 85 years old diagnosed as MGD without atopy, blepharitis, allergic disorders, contact lens, ophthalmic laser treatment, systemic diseases or pregnant	O-3-FA 620 1.5 g per day for 3 months	Placebo 500 mg per day for 3 months	TBUT, Schirmer's test, OSDI score, MG expression, lid margin inflammation, interpalpebral and corneal staining, and adverse events
Sim *et al* [31]; 2014; Singapore	Patients with at least 1/8 eye dry symptoms and at least 1 MG opening with a pouting and a visible plug were recruited; Those with ocular pathology, thyroid dysfunction, rheumatoid arthritis, contact lens wearer or ocular surgery in the past 6 months were excluded	EyeGiene© 10 minutes twice daily for 3 months	Blephasteam© 10 minutes twice daily for 3 months	TBUT, Schirmer's test, MG plugging score, patients compliance and concomitant medications
Yoo *et al* [32]; 2005; Korea	Newly diagnosed patients with MGD with grade 2 or worse destruction or orifice obstruction	Doxycycline 200 mg twice daily for 30 days (although another group in the study had been administered low dose of 20 mg, the data was not considered in the present review)	Placebo twice daily for 30 days	TBUT, Schirmer's test, ocular symptoms
Kashkouli *et al* [33]; 2015; Iran	Patients with posterior blepharitis with no response to conservative management; Those with systemic/topical antibiotics, liver disease, pregnant, lactating mothers, contact lens wearers, allergy to azithromycin or eyelid abnormalities were excluded	500 mg oral azithromycin on day 1 and 250 mg per day from day 2 to day 5	100 mg oral doxycycline twice a day for 1 month	TBUT, MG secretion score, patients symptom score, number of MG plugged orifices, conjuctival injection, lid margin redness and lid margin debris

Suppl. table 1 contd.....

Study id; year; country	Population	Intervention	Comparator	Outcome
Goto *et al* [34]; 2002; Japan	Patients diagnosed as MGD with MG dropout, poor meibum expression and lack of active inflammation; Those with anterior blepharitis of more than moderate severity, infectious conjunctivitis, MGD with acute inflammation, seborrheic MGD and contact lens wearers were excluded	Castor oil 6 times a day for 2 weeks	Placebo 6 times a day for 2 weeks	TBUT, Face score, MG plugging score and tear evaporation rate
Macsai *et al* [35]; 2008; USA	Patients with moderate to severe chronic blepharitis or simple obstructive MGD	O-3-FA 1000 mg three times a day for 12 months	Olive oil three times a day for 12 months	TBUT, MG secretion score, Schirmer test, meibum quality score and MG blockade

© 2017 Sridharan and Sivaramakrishnan.

This is an open access article distributed under the terms of the Creative Commons Attribution 4.0 International Public License (CC-BY 4.0), a copy of which is available at: https://creativecommons.org/licenses/by/4.0/legalcode. This license permits unrestricted use, distribution, and reproduction in any medium, provided the original author and source are credited.
